# Peritumoral *SULF1* Transcript Signal Is Associated with Disease-Specific Survival in Oral Squamous Cell Carcinoma

**DOI:** 10.64898/2026.10.09.26364914

**Published:** 2026-10-12

**Authors:** Gregory Furletti, Trystan Conrad Guerrero, Edward C. Schrom, Bruce Davidson, Bhaskar Kallakury, Radoslav Goldman

## Abstract

The prognostic relevance of peritumoral *SULF1* transcript signal in oral squamous cell carcinoma (OSCC) is unclear. We assembled a retrospective cohort of 133 patients with 218 primary-site tissue-microarray cores; 131 patients had evaluable boundary features. Multiplex fluorescence imaging combined RNAscope *SULF1* , CK-pan, and DAPI. Tumor-stroma boundaries were annotated with the *SULF1* channel hidden. We screened 50 exported boundary features across cumulative stromal bands of 35-300 μm against recurrence-free survival (RFS), overall survival (OS), and disease-specific survival (DSS), with false-discovery-rate correction. The strongest DSS association was signal SD within 150 μm (n = 107; 23 disease-specific deaths; HR = 2.20 per SD; 95% CI, 1.64-2.94; q = 2.1 × 10⁻⁶). In 50 repeated 5-fold cross-validations with feature selection repeated within training folds, adding a boundary feature to AJCC stage increased mean held-out DSS c-index from 0.613 to 0.754 (ΔC = +0.141; 2.5th-97.5th percentiles across repeats, +0.105 to +0.192). These percentiles describe split variability, not confidence intervals. The 150-μm SD feature was selected in 39.2% of DSS training folds. The DSS association persisted after adjustment for mean signal, although the measurements were strongly correlated. Peritumoral *SULF1* signal dispersion was associated with outcome beyond an ordinal AJCC-stage model; independent validation is required.

